# 12-Hydroxyheptadecatrienoic Acid Predicts Hepatocellular Carcinoma Development During Nucleos(t)ide Analogue Therapy

**DOI:** 10.3390/cancers18040542

**Published:** 2026-02-07

**Authors:** Hiroko Ikenaga, Ritsuzo Kozuka, Kirara Inoue, Tsutomu Matsubara, Naoshi Odagiri, Kanako Yoshida, Kohei Kotani, Etsushi Kawamura, Atsushi Hagihara, Hideki Fujii, Masaru Enomoto, Sawako Uchida-Kobayashi

**Affiliations:** 1 Department of Hepatology, Graduate School of Medicine, Osaka Metropolitan University, Osaka 545-8585, Japan; hiroko.ikenaga@omu.ac.jp (H.I.); rkozuka@omu.ac.jp (R.K.); su23017i@st.omu.ac.jp (K.I.); odagiri-n@omu.ac.jp (N.O.); kyoshid@omu.ac.jp (K.Y.); kotanikohei@omu.ac.jp (K.K.); etsushi-k@omu.ac.jp (E.K.); hagy@omu.ac.jp (A.H.); o21717h@omu.ac.jp (H.F.); sawako@omu.ac.jp (S.U.-K.); 2 Department of Biology, Graduate School of Science, Osaka Metropolitan University, Osaka 558-8585, Japan; 3 Department of Anatomy and Regenerative Biology, Graduate School of Medicine, Osaka Metropolitan University, Osaka 545-8585, Japan; matsu335@omu.ac.jp

**Keywords:** biomarker, chronic hepatitis B, fibrosis-4 index, hepatocellular carcinoma, lipidomic analysis, oxylipins, polyunsaturated fatty acids, viral hepatitis

## Abstract

**Background/Objectives**: Alterations in polyunsaturated fatty acid (PUFA) metabolites have been linked to the development of hepatocellular carcinoma (HCC). However, the association between PUFA metabolites and HCC development during nucleos(t)ide analogue (NUC) therapy in patients with chronic hepatitis B virus infection remains unclear. **Methods**: This study enrolled 195 NUC-naïve patients who received NUC therapy. Associations between metabolic factors—especially PUFA metabolites—and HCC development during NUC therapy were evaluated. Baseline serum concentrations of 158 PUFA metabolites were quantified using targeted lipidomic analysis. **Results**: Nineteen patients developed HCC during the follow-up period. The cumulative incidences of HCC at 5 and 10 years were 7.7% and 12.4%, respectively. Variable importance in projection analysis identified 12-hydroxyheptadecatrienoic acid (12-HHT) as the top-ranked metabolite differentiating patients with and without HCC development. Furthermore, 14 metabolites were significantly associated with HCC development based on the log-rank test with 12-HHT being the most significant predictor. The cumulative incidences of HCC at 5 and 10 years were 13.7% and 24.7%, respectively, in patients with 12-HHT concentration ≤ 3.82 ng/mL compared with 3.3% at both time points in those with 12-HHT concentration > 3.82 ng/mL (*p* < 0.001). In multivariate analysis, low 12-HHT concentration (≤3.82 ng/mL; *p* = 0.027; hazard ratio [HR], 4.28; 95% confidence interval [CI], 1.18–15.55) and a fibrosis-4 index ≥ 4.08 (*p* = 0.005; HR, 5.19; 95% CI, 1.64–16.41) were significantly associated with HCC development during NUC therapy. **Conclusions**: Pre-treatment 12-HHT represents a novel predictive biomarker for HCC development during NUC therapy.

## Simple Summary

Even with long-term antiviral therapy for chronic hepatitis B, some patients still develop liver cancer. We investigated whether polyunsaturated fatty acid metabolites—bioactive lipids involved in inflammation and other processes—could predict this risk before antiviral treatment begins. We measured 158 of these metabolites in pre-treatment blood samples from 195 patients starting nucleos(t)ide analogue therapy and followed them. Low levels of 12-hydroxyheptadecatrienoic acid (12-HHT) were strongly linked to liver cancer development. Patients with low 12-HHT had a 4.28-fold higher risk of liver cancer development than those with higher levels. Prediction improved further when 12-HHT was combined with the fibrosis-4 (FIB-4) index, a routine measure of liver scarring. Over 10 years of follow-up, about two-thirds of patients with both high FIB-4 and low 12-HHT developed liver cancer compared with about 1% of those with low FIB-4 and high 12-HHT. If confirmed, this marker could support personalised surveillance and help target prevention during long-term antiviral therapy.

## 1. Introduction

Hepatitis B virus (HBV) infection affects approximately 254 million people worldwide and remains a leading cause of cirrhosis and hepatocellular carcinoma (HCC), resulting in an estimated 1.1 million deaths in 2022 [1].

Currently, nucleos(t)ide analogues (NUCs) including entecavir and tenofovir are widely prescribed for patients with chronic HBV infection [2]. NUC therapy for chronic HBV infection has been shown to suppress viral replication and reduce the risk of HCC development [3,4]. However, some patients develop HCC despite receiving effective NUC therapy. Therefore, identifying risk factors for HCC before initiating NUC therapy is clinically important in patients with chronic HBV infection. Risk factors for HCC development have been broadly classified into host, viral, and environmental categories [5,6,7,8,9,10]. Among environmental factors, metabolic conditions such as obesity and diabetes mellitus have been identified as risk factors for HCC development in patients with chronic HBV infection [3,11,12].

Notably, alterations in lipid metabolites have been closely linked to HCC development among metabolic factors [13]. Previous studies have demonstrated alterations in several lipid metabolites in patients with HBV-related HCC. Du et al. reported decreased levels of several lysophosphatidylcholine species and elevated levels of lysophosphatidic acid species in patients with chronic HBV infection who developed early-stage HCC [14]. Similarly, Abel et al. reported that alterations in membrane cholesterol, phospholipids, and fatty acid profiles are likely to play important roles in HCC progression [15].

Polyunsaturated fatty acids (PUFAs) are important components of cell membranes primarily derived endogenously from phospholipids and play critical roles in various biological processes including both pro- and anti-inflammatory effects [16]. PUFAs are metabolised through cyclooxygenase (COX), lipoxygenase (LOX), and cytochrome P450 (CYP) pathways to produce a broad range of bioactive lipid mediators—prostaglandins, leukotrienes, and epoxyeicosatrienoic acids, respectively [17]. In HCC, alterations in PUFA metabolites have been identified as potential therapeutic targets [18]. Furthermore, lipidomic analyses have identified alterations in patients with HBV-related HCC, suggesting their potential utility as biomarkers [19,20].

However, the association between PUFA metabolites and HCC development during NUC therapy in patients with chronic HBV infection remains unclear. Therefore, we assessed the associations between metabolic factors, especially PUFA metabolites, and HCC development during NUC therapy.

## 2. Materials and Methods

### 2.1. Patients

A total of 195 patients with chronic HBV infection who initiated NUC therapy between September 2006 and July 2023 at Osaka Metropolitan University Hospital. For whom stored serum samples collected before the initiation of therapy were available were included in this retrospective study. Patients who were NUC-naïve and had chronic HBV infection—defined as testing positive for hepatitis B surface antigen (HBsAg) and HBV DNA for at least 6 months before initiating therapy—were treated with entecavir, tenofovir alafenamide, or tenofovir disoproxil fumarate. The inclusion criteria were persistent elevation of serum alanine aminotransferase (ALT) (≥31 U/L) and HBV DNA levels (≥4.0 log copies/mL; equivalent to 3.3 log IU/mL) or advanced fibrosis even when ALT levels were within the normal range in accordance with published guidelines [2,21,22]; absence of clinical signs of HCC before initiating NUC therapy; and no evidence of co-infection with hepatitis C virus, human immunodeficiency virus, or other identifiable causes of chronic liver disease.

This study was conducted in accordance with the principles of the 2013 Declaration of Helsinki. Written informed consent was obtained from all patients before initiation of NUC therapy. The study protocol was approved by the Ethics Committee of Osaka Metropolitan University Hospital (approval numbers 1646, 3260, and 4361).

### 2.2. Study Design

Among the 195 patients, 157 were treated with entecavir, 13 with tenofovir alafenamide, and 25 with tenofovir disoproxil fumarate for more than one year. Entecavir (Baraclude; Bristol-Myers, Tokyo, Japan) was administered orally at a daily dose of 0.5 mg. Tenofovir disoproxil fumarate (Vemlidy; Gilead Sciences, Tokyo, Japan) was administered orally at a daily dose of 25 mg. Tenofovir alafenamide (Tenozet; GlaxoSmithKline, Tokyo, Japan) was administered orally at a daily dose of 300 mg.

Clinical, biochemical, and HBV serological assessments were performed at intervals of one to three months during follow-up. Cirrhosis was diagnosed by histological examination (F4 stage) according to the METAVIR scoring system [23] supported by imaging findings from ultrasonography, computed tomography (CT), or magnetic resonance imaging (MRI), and by the presence of portal hypertension defined by clinical features such as oesophageal or gastric varices or ascites. Steatotic liver disease (SLD) was diagnosed by trained sonographers based on ultrasonographic findings including a bright liver, hepatorenal echo contrast, deep attenuation, and vessel blurring [24].

### 2.3. Hepatocellular Carcinoma Surveillance

The study endpoint was HCC development during NUC therapy. Patients who developed HCC within one year after initiating NUC therapy were excluded. All patients underwent ultrasonography or dynamic CT or MRI every 3–6 months for HCC surveillance. HCC was diagnosed by percutaneous needle biopsy or by characteristic imaging findings of arterial phase hyperenhancement and delayed washout on dynamic CT or MRI. Patients were followed up until the diagnosis of HCC was confirmed or until their last clinical visit before October 2024.

### 2.4. Laboratory Assays

Complete blood counts and serum measurements of aspartate aminotransferase (AST), ALT, gamma-glutamyl transferase, total bilirubin, and albumin levels were obtained using standard laboratory procedures. Serum α-fetoprotein (AFP) concentrations were determined using a chemiluminescent enzyme immunoassay. The fibrosis-4 (FIB-4) index was calculated using Sterling’s formula: age (years) × AST (IU/L)/[platelet count (×10^9^/L) × √ALT (IU/L)]. Hepatitis B core-related antigen (HBcrAg) was quantified using a novel ultrasensitive assay, the “immunoassay for total antigen including complex via pre-treatment” (Fuji-Rebio, Tokyo, Japan) [9]. HBsAg was quantified using a chemiluminescent microparticle immunoassay (Architect HBsAg QT; Abbott Japan Corp., Tokyo, Japan). HBV DNA was quantified by real-time polymerase chain reaction assay (COBAS TaqMan HBV Test, ver. 2.0; Roche Diagnostics K.K., Tokyo, Japan). HBV genotype was determined using an enzyme-linked immunosorbent assay employing monoclonal antibodies specific to epitopes in the preS2 region (Institute of Immunology, Tokyo, Japan).

### 2.5. Analysis of the Serum Lipidome

Serum samples (30 μL) were diluted with 300 μL of 0.1% formic acid (Fujifilm Wako, Osaka, Japan; 067-04531) in methanol (Fujifilm Wako; 138-14521) containing an internal standard mixture of 10 ng/mL prostaglandin (PG) E2-d4 (Cayman Chemical, Ann Arbor, MI, USA; 314010), 10 ng/mL leukotriene (LT) B4-d4 (Cayman Chemical; 320110), and 100 ng/mL arachidonic acid (AA)-d8 (Cayman Chemical; 390010). The mixture was then centrifuged at 15,000× *g* for 10 min to remove insoluble materials. The resulting supernatant was loaded onto Strata-X extraction cartridges (Phenomenex, Torrance, CA, USA; 8B-S100-AAK) for purification, evaporated using a centrifugal evaporator (SpeedVac, Thermo Fisher Scientific, Waltham, MA, USA), and reconstituted in 30 μL of methanol. Finally, 5 μL of each of the reconstituted samples was analysed using an LC/MS system (LCMS-8060, Shimadzu, Kyoto, Japan) comprising a Nexera^TM^ X2 unit (Shimazu) coupled to a mass spectrometer (MS). The concentrations of 158 polyunsaturated fatty acid metabolites were estimated using a triple quadrupole MS (Shimadzu) and the LC/MS/MS Method Package for Lipid Mediators Ver. 2 (Shimadzu). Peak deconvolution was performed using the Traverse MS software, version 1.2.9 (Reifycs Inc., Tokyo, Japan). In addition, 12-hydroxyheptadecatrienoic acid (12[S]-HHTrE [12-HHT] [Cayman Chemical; 34590]) was quantified using a calibration curve.

### 2.6. Statistical Analysis

Statistical analyses were performed using R statistical software, version 4.2.3. Baseline characteristics between groups were compared using the χ^2^ test for categorical variables and the Mann–Whitney U test for continuous variables. Receiver operator curves were generated for each variable to determine optimal cut-off values distinguishing patients with and without HCC during NUC therapy. Multivariate analyses were performed using the MetaboAnalyst 6.0 platform (www.metaboanalyst.ca (accessed on 10 August 2025)). Metabolite peak areas were log_2_-transformed and auto-scaled for normalisation. Partial least squares discriminant analysis (PLS-DA) was conducted to visualise the separation between patients with and without HCC development. Variable importance in projection (VIP) scores were used to identify metabolites contributing to group separation. Kaplan–Meier analysis and the log-rank test were used to estimate and compare cumulative incidences of HCC development between the two groups. *p* values were adjusted for multiple comparisons using the Benjamini–Hochberg false discovery rate procedure. Cox proportional hazard models were applied to analyse factors associated with HCC development. Correlation significance was evaluated by Spearman’s rank analysis. All reported *p*-values were two-sided, with statistical significance set at *p*-value < 0.05.

## 3. Results

### 3.1. Baseline Characteristics of the Patients

Baseline patient characteristics are summarised in Table 1. The median age of the patients was 46.0 years (interquartile range [IQR], 39.0, 56.0). The cohort included 117 (60.0%) males, 31 (15.9%) with cirrhosis, 12 (6.2%) with diabetes, and 58 (29.7%) with SLD. A total of 167 patients (85.6%) had HBV genotype C. The median follow-up duration was 7.4 years (range: 1.0–17.9).

### 3.2. Cumulative Rates of HCC Development According to Clinical Factors at Baseline

**Figure A1:**
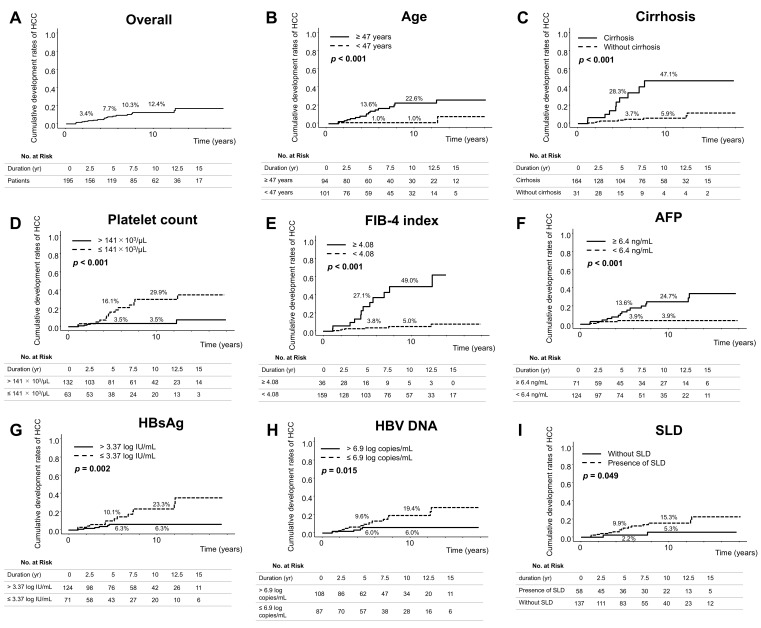
Cumulative rates of HCC development during NUC therapy in (**A**) all patients and according to (**B**) age, (**C**) cirrhosis, (**D**) platelet count, (**E**) FIB-4 index, (**F**) AFP, (**G**) HBsAg, (**H**) HBV DNA, and (**I**) SLD at baseline. AFP, α-fetoprotein; FIB-4, fibrosis-4 index; HBsAg, hepatitis B surface antigen; HBV, hepatitis B virus; HCC, hepatocellular carcinoma; NUC, nucleos(t)ide analogue; SLD, steatotic liver disease.

During follow-up, 19 patients developed HCC, with a median duration, 4.3 years (range, 1.1–12.4). The cumulative incidence of HCC at 5 and 10 years were 7.7% and 12.4%, respectively (Figure A1A). Based on log-rank testing, age ≥ 47 years (*p* < 0.001), cirrhosis (*p* < 0.001), platelet count ≤ 141 × 10^3^/μL (*p* < 0.001), FIB-4 index ≥ 4.08 (*p* < 0.001), AFP ≥ 6.4 ng/mL (*p* < 0.001), HBsAg ≤ 3.37 log IU/mL (*p* = 0.002), HBV-DNA ≤ 6.9 log copies/mL (*p* = 0.015), and SLD (*p* = 0.049) at baseline were significantly associated with HCC development (Figure A1B–I).

### 3.3. Metabolites at Baseline Associated with Hepatocellular Carcinoma Development During NUC Therapy

**Figure 1:**
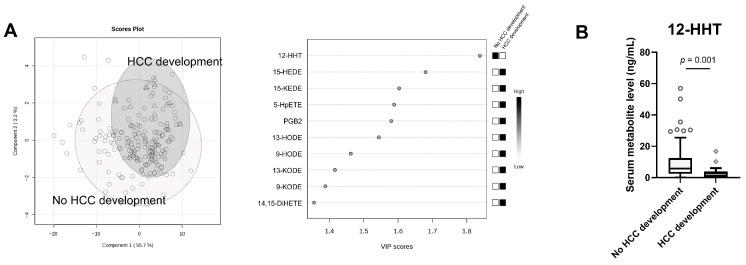
(**A**) PLS-DA score plots (**left**) and metabolites with the highest VIP scores (**right**) to distinguish patients with and without HCC development during NUC therapy. Component 1 and 2 represent the first and second latent components of the PLS-DA model. The percentages on each axis indicate the proportion of variance in the metabolite data explained by each component. Each data point represents an individual patient sample. Different point shapes are used only to distinguish between patient groups. (**B**) Serum 12-HHT levels at baseline between patients with and without HCC development. 12-HHT, 12-hydroxyheptadecatrienoic acid; DiHETE, dihydroxy-eicosatetraenoic acid; HCC, hepatocellular carcinoma; HEDE, hydroxy-eicosadienoic acid; HODE, hydroxy-octadecadienoic acid; HpETE, hydroperoxy-eicosatetraenoic acid; KEDE, 15-oxo-11Z,13E-eicosadienoic acid; KODE, keto-octadecadienoic acid; NUC, nucleos(t)ide analogue; PG, prostaglandin; PLS-DA, partial least squares discriminant analysis; VIP, variable importance in projection.

Among 158 PUFA metabolites measured, 76 were detected in serum and included in the analysis. Score plots based on metabolite profiles were generated separately for patients with and without HCC development. The VIP analysis identified 12-HHT as the highest-ranked metabolite (VIP score = 1.84), differentiating patients with and without HCC development (Figure 1A). Serum 12-HHT concentrations were significantly lower in patients with HCC development (*p* = 0.001) (Figure 1B).

**Figure 2:**
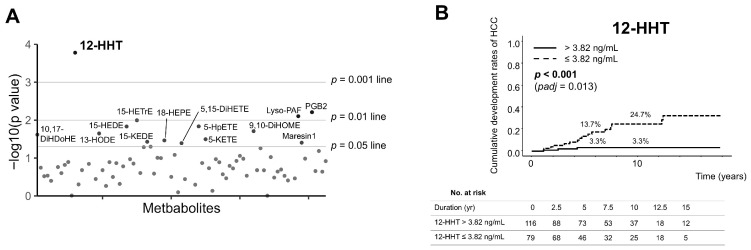
(**A**) Statistical results of the log-rank test for HCC development during NUC therapy based on binary groups defined by ROC-derived cut-off values from MS measurements. The y-axis represents the *p*-value (−log_10_ scale). *p* value was calculated by log-rank test. (**B**) Cumulative rates of HCC development according to serum quantitative 12-HHT levels. Adjusted *p* values were calculated using the Benjamini–Hochberg false discovery rate (FDR) procedure. “No. at risk” indicates the number of patients at risk of HCC development at each time point. 12-HHT, 12-hydroxyheptadecatrienoic acid; DiHDoHE, dihydroxy-docosahexaenoic acid; DiHETE, dihydroxy-eicosatetraenoic acid; DiHOME, dihydroxyoctadecenoic acid; HCC, hepatocellular carcinoma; HEDE, hydroxy-eicosadienoic acid; HEPE, hydroxy-eicosapentaenoic acid; HETrE, hydroxy-eicosatrienoic acid; HODE, hydroxy-octadecadienoic acid; HpETE, hydroperoxy-eicosatetraenoic acid; KEDE, oxoeicosadienoic acid; KETE, oxoeicosatetraenoic acid; Lyso-PAF, lyso-platelet activating factor; NUC, nucleos(t)ide analogue; PGB2, prostaglandin B2; ROC receiver operating characteristic.

**Figure A2:**
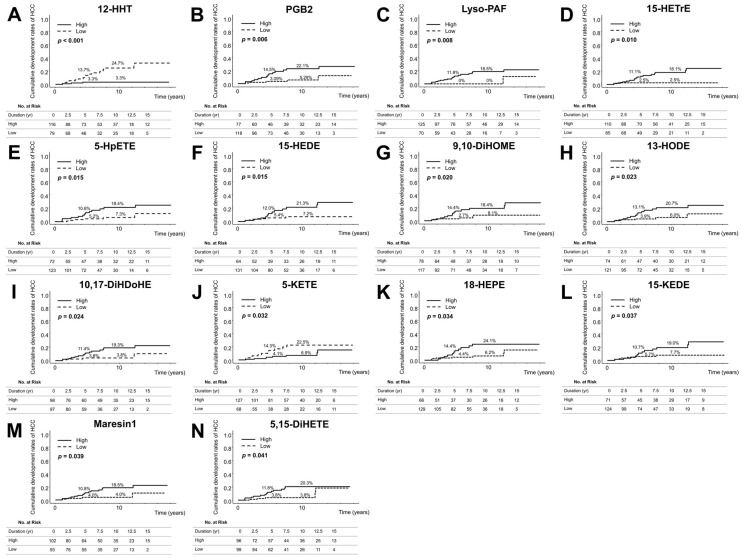
Cumulative rates of HCC development during NUC therapy according to the serum (**A**) 12-HHT, (**B**) PGB2, (**C**) Lyso-PAF, (**D**) 15-HETrE, (**E**) 5-HpETE, (**F**) 15-HEDE, (**G**) 9,10-DiHOME, (**H**) 13-HODE, (**I**) 10,17-DiHDoHE, (**J**) 5-KETE, (**K**) 18-HEPE, (**L**) 15-KEDE, (**M**) Maresin1, and (**N**) 5,15-DiHETE levels. Low and high groups were classified based on ROC-derived cut-off values from MS measurements and *p*-values were calculated using the log-rank test. 12-HHT, 12-hydroxyheptadecatrienoic acid; DiHDoHE, dihydroxy-docosahexaenoic acid; DiHETE, dihydroxy-eicosatetraenoic acid; DiHOME, dihydroxyoctadecenoic acid; HCC, hepatocellular carcinoma; HEDE, hydroxy-eicosadienoic acid; HEPE, hydroxy-eicosapentaenoic acid; HETrE, hydroxy-eicosatrienoic acid; HODE, hydroxy-octadecadienoic acid; HpETE, hydroperoxy-eicosatetraenoic acid; KEDE, oxoeicosadienoic acid; KETE, oxoeicosatetraenoic acid; Lyso-PAF, lyso-platelet activating factor; NUC, nucleos(t)ide analogue; PG, prostaglandin; ROC, receiver operating characteristic.

According to the log-rank test, 14 metabolites were significantly associated with HCC development with 12-HHT showing the smallest *p*-value (Figure 2A and Figure A2). We additionally calculated false discovery rate (FDR)-adjusted *p* values using the Benjamini–Hochberg procedure (*q* values) and only 12-HHT remained statistically significant (*q* = 0.013). The cumulative rates of HCC development at 5 and 10 years were 13.7% and 24.7%, respectively, among patients with 12-HHT levels ≤ 3.82 ng/mL, and 3.3% at both time points among those with 12-HHT levels > 3.82 ng/mL (*p* < 0.001) (Figure 2B).

### 3.4. Correlations Between PUFA Metabolites and Age, FIB-4 Index, or α-Fetoprotein Levels at Baseline

**Figure A3:**
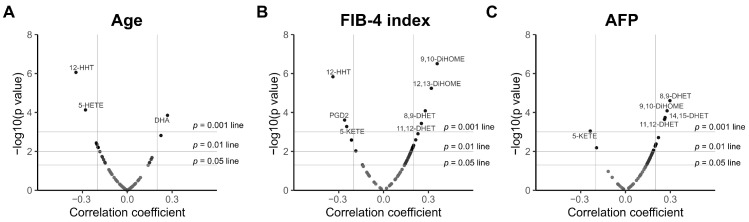
Correlations between PUFA metabolites and (**A**) age, (**B**) FIB-4 index, and (**C**) AFP at baseline. Spearman’s correlation analysis was conducted to identify metabolites with particularly strong associations, and those with *p* < 0.001 and an absolute Spearman’s correlation coefficient (|r|) > 0.2 were selected. 12-HHT, 12-hydroxyheptadecatrienoic acid; AFP, α-fetoprotein; DHA, docosahexaenoic acid; DHET, dihydroxyeicosatrienoic acid; DiHOME, dihydroxyoctadecenoic acid; FIB-4, fibrosis-4 index; HETE, hydroxyeicosatetraenoic acid; KETE, keto-eicosatetraenoic acid; PG, prostaglandin; PUFA, polyunsaturated fatty acid.

12-HHT and 5-hydroxyeicosatetraenoic acid (HETE) showed negative correlation with age, whereas docosahexaenoic acid (DHA) showed positive correlation (Figure A3A). 12-HHT, PGD2, and 5-ketoeicosatetraenoic acid (5-KETE) showed negative correlation with the FIB-4 index, whereas 9,10-dihydroxyoctadecenoic acid (9, 10-DiHOME), 12,13-DiHOME, 8,9-dihydroxyeicosatrienoic acid (8,9-DHET), and 11,12-DHET showed positive correlation (Figure A3B). 5-KETE showed negative correlation with AFP, whereas 8,9-DHET, 9,10-DiHOME, 14,15-DHET, and 11,12-DHET showed positive correlation (Figure A3C).

### 3.5. Factors at Baseline Predicting Hepatocellular Carcinoma Development During NUC Therapy

Univariate analysis identified several baseline factors predicting HCC development during NUC therapy including 12-HHT ≤ 3.82 ng/mL (*p* = 0.001; hazard ratio [HR], 7.51; 95% confidence interval [CI], 2.19–25.82), age ≥ 47 years (*p* = 0.005; HR, 8.24; 95% CI, 1.90–35.70), cirrhosis (*p* < 0.001; HR, 7.03; 95% CI, 2.84–17.38), platelet count ≤ 141 × 10^3^/μL (*p* < 0.001; HR, 5.88; 95% CI, 2.11–16.33), FIB-4 index ≥ 4.08 (*p* < 0.001; HR, 10.41; 95% CI, 4.07–26.60), AFP level ≥ 6.4 ng/mL (*p* = 0.001; HR, 6.10; 95% CI, 2.02–18.38), HBsAg ≤ 3.37 log IU/mL (*p* = 0.005; HR, 4.01; 95% CI, 1.52–10.55), and HBV DNA ≤ 6.9 log copies/mL (*p* = 0.022; HR, 3.29; 95% CI, 1.19–9.14).

Multivariate analysis revealed that 12-HHT ≤ 3.82 ng/mL (*p* = 0.027; HR, 4.28; 95% CI, 1.18–15.55) and FIB-4 index ≥ 4.08 (*p* = 0.005; HR, 5.19; 95% CI, 1.64–16.41) were independent factors significantly associated with HCC development during NUC therapy (Table 2). Other multivariable analyses adjusted for 12-HHT and AFP are presented in Table A1.

### 3.6. Correlation Between 12-Hydroxyheptadecatrienoic Acid and Clinical Factors at Baseline

**Figure 3:**
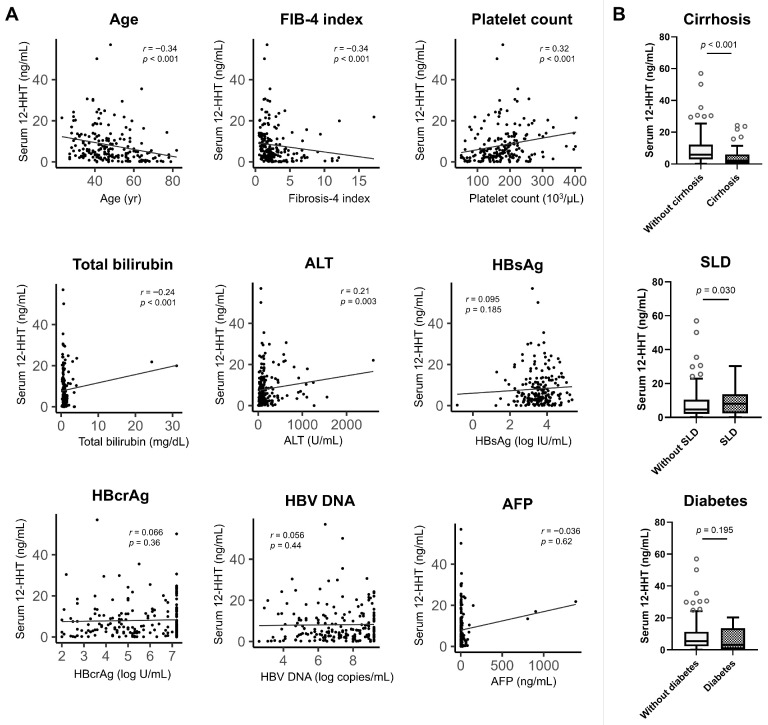
(**A**) Correlations between 12-HHT and clinical factors (age, FIB-4 index, platelet count, total bilirubin, ALT, HBsAg, HBcrAg, HBV DNA, and AFP). (**B**) Serum 12-HHT levels at baseline between patients with and without cirrhosis, SLD, or diabetes. 12-HHT, 12-hydroxyheptadecatrienoic acid; AFP, α-fetoprotein; ALT, alanine aminotransferase; FIB-4 index, fibrosis-4 index; HBcrAg, hepatitis B core-related antigen; HBsAg, hepatitis B surface antigen; HBV, hepatitis B virus; SLD, steatotic liver disease.

Baseline characteristics of patients with 12-HHT ≤ 3.82 ng/mL and those with 12-HHT > 3.82 ng/mL are presented in Table 3. 12-HHT levels showed negative correlations with age (Spearman’s correlation coefficient *r* = −0.34, *p* < 0.001), the FIB-4 index (*r* = −0.34, *p* < 0.001), and total bilirubin (*r* = −0.24, *p* < 0.001), and positive correlation with platelet count (*r* = 0.32, *p* < 0.001) and ALT (*r* = 0.21, *p* = 0.003) (Figure 3A). 12-HHT levels were significantly decreased in patients with cirrhosis (*p* < 0.001) and those with SLD (*p* = 0.030) (Figure 3B).

### 3.7. Cumulative Rates of the Hepatocellular Carcinoma Development According to the Combination of FIB-4 Index and 12-HHT at Baseline After NUC Therapy

**Figure 4:**
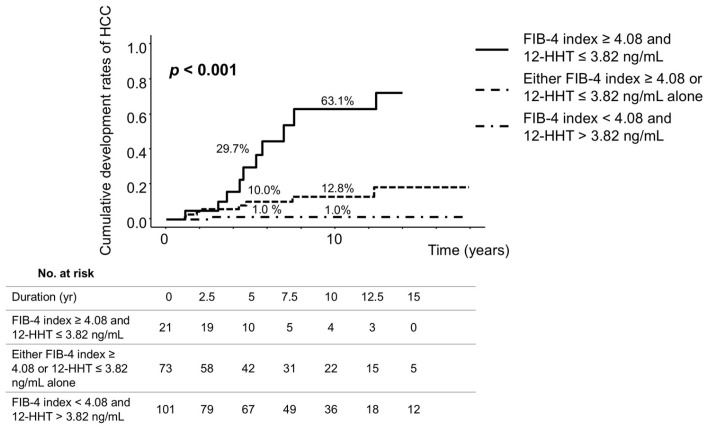
Cumulative rates of HCC development based on the combination of FIB-4 index and 12-HHT at baseline after NUC therapy. Patients were classified into three groups based on the FIB-4 index and 12-HHT levels at baseline after NUC therapy, using cut-off values of 4.08 and 3.82 ng/mL, respectively, to distinguish between patients with and without HCC during NUC therapy. “No. at risk” indicates the number of patients at risk of HCC development at each time point. 12-HHT, 12-hydroxyheptadecatrienoic acid; FIB-4 index, fibrosis-4 index; HCC, hepatocellular carcinoma; NUC, nucleos(t)ide analogue.

Patients were classified into three groups according to the baseline FIB-4 index and 12-HHT levels after NUC therapy using cut-off values of 4.08 and 3.82 ng/mL, respectively, to distinguish between patients with and without HCC during NUC therapy. The 5- and 10-year cumulative rates of HCC development were 29.7% and 63.1%, respectively, among patients with both FIB-4 index ≥ 4.08 and 12-HHT ≤ 3.82 ng/mL (n = 21); 10.0% and 12.8%, respectively, among those with either FIB-4 index ≥ 4.08 or 12-HHT ≤ 3.82 ng/mL alone (n = 73); and 1.0% at both time points among patients with FIB-4 index < 4.08 and 12-HHT > 3.82 ng/mL (n = 101) (*p* < 0.001) (Figure 4).

## 4. Discussion

As far as we know, this study is the first report that evaluates the association between PUFA metabolites and HCC development during NUC therapy in patients with chronic HBV infection using targeted lipidomic analysis. In the present analysis, baseline serum PUFA metabolite levels were measured in patients receiving NUC therapy. Our findings indicate that pre-treatment levels of 12-HHT and FIB-4 index are useful predictors of HCC development during NUC therapy and that predictive accuracy improves when the two are combined.

Altered lipid metabolism represents one of the most prominent metabolic changes observed in cancer [25]. The eicosanoid pathway, which generates PUFA metabolites, is regarded as a key pathway associated with liver inflammation and carcinogenesis [18,26]. PUFA metabolites are derived from PUFAs including arachidonic acid, DHA, and eicosapentaenoic acid through COX, LOX, or CYP pathways [16]. Several of these metabolites have been implicated in carcinogenesis. For instance, PGE_2_ has been shown to promote cancer progression [27,28]. The LOX family has also been suggested to contribute, at least in part, to HCC development [29,30,31].

In the context of clinical biomarker research, lipidomic analyses have been conducted to identify PUFA metabolites associated with HCC. Gong et al. performed a metabolomic analysis that included several PUFA metabolites and reported significantly higher serum levels of PGF_2_α, thromboxane (TX)B_2_, 5-HETE, and 15-HETE in patients with HCC compared with those with chronic HBV infection [19]. Similarly, Lu et al. demonstrated that serum levels of 9,10-DiHOME and 12,13-DiHOME were higher in patients with HBV-related HCC than in those with chronic hepatitis [20]. However, these studies neither adjusted for clinical confounders nor assessed the long-term risk of HCC development during NUC therapy. In this study, we conducted targeted lipidomic analysis in patients treated with NUC and evaluated the predictive value of PUFA metabolites for HCC development during NUC therapy using both univariate and multivariate analyses. We identified 14 metabolites that may serve as novel prognostic biomarkers for HCC with 12-HHT emerging as the strongest independent predictor of HCC development.

**Figure 5:**
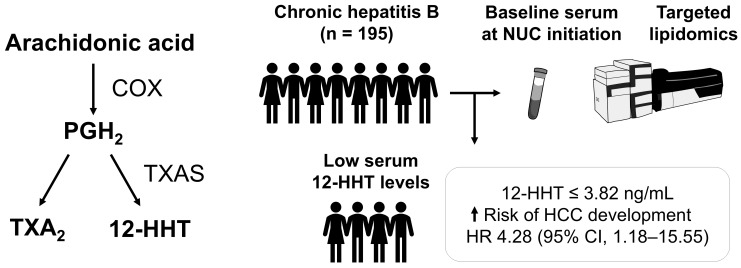
Low baseline serum 12-HHT (≤3.82 ng/mL) was associated with an increased risk of hepatocellular carcinoma development during nucleos(t)ide analogue therapy. 12-HHT, 12-hydroxyheptadecatrienoic acid; COX, cyclooxygenase; HCC, hepatocellular carcinoma; NUC, nucleos(t)ide analogue; PGH_2_, prostaglandin H_2_; TXA_2_, thromboxane A_2_; TXAS, thromboxane synthase.

12-HHT is a 17-carbon hydroxy fatty acid biosynthesised either through enzymatic pathways including thromboxane synthase (TXAS) and COX, or via non-enzymatic processes [32]. TXAS catalyses the isomerisation of PGH_2_ into 12-HHT and TXA_2_ (Figure 5). Traditionally, 12-HHT was considered a byproduct of TXA_2_ biosynthesis and its biological role remained unclear. However, several studies have demonstrated that 12-HHT acts as an endogenous agonist of leukotriene B4 receptor 2 (BLT2) [33,34]. The 12-HHT–BLT2 axis has been implicated in wound healing [35] and evidence suggests that this interaction may also enhance intestinal barrier function [36,37,38]. Emerging evidence suggests that alterations in gut microbiota and intestinal barrier dysfunction are strongly associated with HCC development [39,40,41]. Reduced levels of 12-HHT in patients with HBV infection may therefore contribute to intestinal barrier impairment and promote HCC progression. Further studies are required to elucidate the mechanisms through which 12-HHT may exert tumour-suppressive effects.

The FIB-4 index is well established as a surrogate marker of liver fibrosis and a predictor of HCC development [42]. Several studies have shown that an elevated baseline FIB-4 index is strongly associated with HCC development in patients undergoing NUC therapy [43,44], findings that are consistent with our results. Furthermore, combining the FIB-4 index with other clinical and serological markers such as AFP or novel biomarkers enhances predictive performance. Notably, 12-HHT emerged as a strong independent predictor in this study and the combination of 12-HHT with the FIB-4 index further enhanced risk stratification during NUC therapy.

The present study has some limitations. First, it was conducted at a single centre with a relatively small sample size. Second, the potential mechanisms by which 12-HHT may contribute to carcinogenesis were not investigated. Third, given the large number of metabolites analysed, the possibility of multiple testing cannot be excluded. This concern was addressed by applying PLS-DA, which integrates all variables into a single multivariate model and thereby reduces the risk of false-positive findings.

## 5. Conclusions

In summary, low baseline serum levels of 12-HHT are strongly associated with an increased risk of HCC in patients undergoing NUC therapy. Moreover, combining 12-HHT with the FIB-4 index markedly enhanced risk stratification. These findings suggest that pre-treatment 12-HHT is a novel predictive biomarker for HCC development in patients undergoing NUC therapy.

## Figures and Tables

**Table 1:** Baseline characteristics of the patients.

Variables	Total (n = 195)	No HCC Development (n = 176)	HCC Development (n = 19)	*p* Value
Age (yr)	46.0 [39.0, 56.0]	44.5 [37.8, 56.0]	55.0 [48.0, 62.5]	0.002 **
Sex: Male	117 (60.0)	103 (58.5)	14 (73.7)	0.20
BMI (kg/m^2^)	22.4 [20.8, 25.0]	22.4 [20.5, 24.9]	22.5 [21.9, 25.7]	0.23
Cirrhosis	31 (15.9)	21 (11.9)	10 (52.6)	<0.001 ***
Alcohol intake	27 (13.8)	23 (13.1)	4 (21.1)	0.34
Diabetes	12 (6.2)	9 (5.1)	3 (15.8)	0.079
Steatotic liver disease	58 (29.7)	56 (31.8)	2 (10.5)	0.054
Platelet count (10^3^/μL)	173 [134, 214]	181 [138, 216]	115 [72, 151]	<0.001 ***
FIB-4 index	1.99 [1.33, 3.28]	1.90 [1.29, 2.99]	4.25 [2.37, 6.74]	<0.001 ***
AST (U/L)	66 [42, 122]	63 [42, 125]	67 [45, 91]	0.80
ALT (U/L)	87 [54, 201]	94 [54, 206]	74 [52, 117]	0.26
GGT (U/L)	46 [26, 95]	45 [25, 92]	57 [37, 112]	0.22
Total bilirubin (mg/dL)	0.80 [0.60, 1.05]	0.80 [0.60, 1.00]	0.90 [0.70, 1.15]	0.36
Albumin (g/dL)	4.10 [3.80, 4.30]	4.10 [3.80, 4.40]	4.00 [3.80, 4.10]	0.122
AFP (ng/mL)	4.6 [2.9, 10]	4.5 [2.7, 8.2]	10.9 [6.5, 29.9]	<0.001 ***
HBsAg (log IU/mL)	3.55 [3.08, 4.03]	3.64 [3.17, 4.06]	3.03 [2.86, 3.52]	0.001 **
HBcrAg (log U/mL)	5.90 [4.40, ≥7.1]	6.10 [4.40, ≥7.1]	5.30 [4.60, 6.05]	0.140
HBV DNA (log copies/mL)	7.30 [6.00, 8.70]	7.40 [6.10, 8.80]	6.20 [5.35, 7.20]	0.010 *

Values are reported as n (%) or median [IQR]. * *p* < 0.05, ** *p* < 0.01, ***, *p* < 0.001. AFP, α-fetoprotein; ALT, alanine aminotransferase; AST, aspartate aminotransferase; BMI, body mass index; FIB-4 index, fibrosis-4 index; GGT, gamma-glutamyl transferase; HBcrAg, hepatitis B core-related antigen; HBsAg, hepatitis B surface antigen; HBV, hepatitis B virus; IQR, interquartile range.

**Table 2:** Factors at baseline predicting the development of HCC during NUC therapy.

		Univariate Analysis		Multivariate Analysis	
Variables	Category	HR (95% CI)	*p* Value	HR (95% CI)	*p* Value
12-HHT	≤3.82 ng/mL	7.51 (2.19–25.82)	0.001 **	4.28 (1.18–15.55)	0.027 *
Age	≥47 years	8.24 (1.90–35.70)	0.005 **		
Sex	Male	1.69 (0.61–4.69)	0.32		
Liver fibrosis	Cirrhosis	7.03 (2.84–17.38)	<0.001 ***	1.84 (0.60–5.65)	0.28
Diabetes	Diabetes	2.68 (0.78–9.25)	0.118		
Alcohol intake	Drinker	1.53 (0.51–4.60)	0.45		
SLD	(+)	0.26 (0.06–1.11)	0.068		
Platelet count	≤141 × 10^3^/μL	5.88 (2.11–16.33)	<0.001 ***		
FIB-4 index	≥4.08	10.41 (4.07–26.60)	<0.001 ***	5.19 (1.64–16.41)	0.005 **
AST	≥80 U/L	1.19 (0.48–2.97)	0.70		
ALT	≤81 U/L	2.04 (0.80–5.20)	0.133		
GGT	≥55 U/L	1.81 (0.73–4.51)	0.20		
Total bilirubin	≥1.0 mg/dL	2.05 (0.83–5.06)	0.110		
Albumin	≤4.0 g/L	2.41 (0.91–6.34)	0.075		
AFP	≥6.4 ng/mL	6.10 (2.02–18.38)	0.001 **		
HBsAg	≤3.37 log IU/mL	4.01 (1.52 –10.55)	0.005 **		
HBV-DNA	≤6.9 log copies/mL	3.29 (1.19–9.14)	0.022 *		
HBcrAg	≤5.9 log U/mL	2.51 (0.90–6.97)	0.077		

* *p* < 0.05, ** *p* < 0.01, ***, *p* < 0.001. 12-HHT, 12-hydroxyheptadecatrienoic acid; AFP, α-fetoprotein; ALT, alanine aminotransferase; AST, aspartate aminotransferase; CI, confidence interval; FIB-4 index, fibrosis-4 index; GGT, gamma-glutamyl transferase; HBcrAg, hepatitis B core-related antigen; HBsAg, hepatitis B surface antigen; HBV, hepatitis B virus; HCC, hepatocellular carcinoma; HR, hazard ratio; NUC, nucleos(t)ide analogue; SLD, steatotic liver disease.

**Table 3:** Factors at baseline predicting the development of HCC during NUC therapy.

Variables	Low 12-HHT (n = 79)	High 12-HHT (n = 116)	*p* Value
Age (yr)	52.0 [42.5, 63.0]	42.5 [37.0, 50.3]	<0.001 ***
Sex: Male	47 (59.5)	70 (60.3)	0.91
BMI (kg/m^2^)	22.4 [20.5, 24.8]	22.4 [20.9, 25.1]	0.49
Cirrhosis	22 (27.8)	9 (7.8)	<0.001 ***
Alcohol intake	11 (13.9)	16 (13.8)	0.98
Diabetes	8 (10.1)	4 (3.4)	0.057
SLD	17 (21.5)	41 (35.3)	0.038 *
Platelet count (10^3^/μL)	148 [111, 200]	188 [157, 220]	<0.001 ***
FIB-4 index	2.41 [1.78, 4.11]	1.75 [1.12, 2.84]	<0.001 ***
AST (U/L)	66 [41, 104]	65 [45, 130]	0.23
ALT (U/L)	72 [47, 158]	113 [60, 254]	0.017 *
GGT (U/L)	40 [24, 77]	53 [28, 99]	0.099
Total bilirubin (mg/dL)	0.90 [0.70, 1.20]	0.70 [0.50, 1.00]	0.004 **
Albumin (g/dL)	4.00 [3.80, 4.30]	4.15 [3.90, 4.40]	0.151
AFP (ng/mL)	4.90 [2.90, 11.05]	4.55 [2.88, 8.57]	0.54
HBsAg (log IU/mL)	3.53 [3.00, 3.94]	3.68 [3.21, 4.07]	0.074
HBcrAg (log U/mL)	5.40 [4.30, ≥7.1]	6.10 [4.50, ≥7.1]	0.25
HBV DNA (log copies/mL)	7.20 [5.85, 8.55]	7.40 [6.00, 8.72]	0.42

Values are reported as n (%) or median [IQR]. * *p* < 0.05, ** *p* < 0.01, *** *p* < 0.001. Low 12-HHT was defined as serum 12-HHT levels ≤ 3.82 ng/mL, and high 12-HHT as >3.82 ng/mL. 12-HHT, 12-hydroxyheptadecatrienoic acid; AFP, α-fetoprotein; ALT, alanine aminotransferase; AST, aspartate aminotransferase; BMI, body mass index; FIB-4 index, fibrosis-4 index; GGT, gamma-glutamyl transferase; HBcrAg, hepatitis B core-related antigen; HBsAg, hepatitis B surface antigen; HBV, hepatitis B virus; IQR, interquartile range; SLD, steatotic liver disease.

## Data Availability

The datasets generated and analysed during the current study are not publicly available due to patient confidentiality and ethical restrictions but are available from the corresponding author on reasonable request and with appropriate institutional approval.

## Abbreviations

The following abbreviations are used in this manuscript:

12-HHT	12-hydroxyheptadecatrienoic acid
AA	arachidonic acid
AFP	α-fetoprotein
ALT	alanine aminotransferase
AST	aspartate aminotransferase
BLT2	leukotriene B4 receptor 2
CI	confidence interval
COX	cyclooxygenase
CT	computed tomography
CYP	cytochrome P450
DHA	docosahexaenoic acid
DHET	dihydroxyeicosatrienoic acid
DiHOME	dihydroxyoctadecenoic acid
FIB-4	fibrosis-4
HBcrAg	hepatitis B core-related antigen
HBsAg	hepatitis B surface antigen
HBV	hepatitis B virus
HCC	hepatocellular carcinoma
HETE	hydroxyeicosatetraenoic acid
HR	hazard ratio
IQR	interquartile range
KETE	ketoeicosatetraenoic acid
LOX	lipoxygenase
LT	leukotriene
MRI	magnetic resonance imaging
MS	mass spectrometry
NUC	nucleos(t)ide analogue
PG	prostaglandin
PLS-DA	partial least squares discriminant analysis
PUFA	polyunsaturated fatty acid
SLD	steatotic liver disease
TX	thromboxane
TXAS	thromboxane synthase
VIP	variable importance in projection

## Appendix A

**Table A1:** Multivariable Cox proportional hazards models including 12-HHT and AFP for the development of HCC during NUC therapy.

		Multivariate Analysis	
Variables	Category	HR (95% CI)	*p* Value
12-HHT	≤3.82 ng/mL	7.08 (2.06–24.34)	0.002 **
AFP	≥6.4 ng/mL	5.73 (1.90–17.27)	0.002 **

** *p* < 0.01. 12-HHT, 12-hydroxyheptadecatrienoic acid; AFP, α-fetoprotein; CI, confidence interval; HCC, hepatocellular carcinoma; HR, hazard ratio; NUC, nucleos(t)ide analogue.

## References

[1] World Health Organization (2025). Hepatitis B.

[2] Asahina Y. (2020). Drafting Committee for Hepatitis Management Guidelines, the Japan Society of Hepatology Japan Society of Hepatology Guidelines for the Management of Hepatitis B Virus Infection: 2019 Update. Hepatol. Res..

[3] Wu C.-Y., Lin J.-T., Ho H.J., Su C.-W., Lee T.-Y., Wang S.-Y., Wu C., Wu J.-C. (2014). Association of Nucleos(t)Ide Analogue Therapy with Reduced Risk of Hepatocellular Carcinoma in Patients with Chronic Hepatitis B: A Nationwide Cohort Study. Gastroenterology.

[4] Singal A.K., Salameh H., Kuo Y.-F., Fontana R.J. (2013). Meta-Analysis: The Impact of Oral Anti-Viral Agents on the Incidence of Hepatocellular Carcinoma in Chronic Hepatitis B. Aliment. Pharmacol. Ther..

[5] Kumada T., Toyoda H., Tada T., Kiriyama S., Tanikawa M., Hisanaga Y., Kanamori A., Niinomi T., Yasuda S., Andou Y. (2013). Effect of Nucleos(t)Ide Analogue Therapy on Hepatocarcinogenesis in Chronic Hepatitis B Patients: A Propensity Score Analysis. J. Hepatol..

[6] Yamada R., Hiramatsu N., Oze T., Morishita N., Harada N., Yakushijin T., Iio S., Doi Y., Yamada A., Kaneko A. (2015). Impact of Alpha-Fetoprotein on Hepatocellular Carcinoma Development during Entecavir Treatment of Chronic Hepatitis B Virus Infection. J. Gastroenterol..

[7] Kozuka R., Enomoto M., Sato-Matsubara M., Yoshida K., Motoyama H., Hagihara A., Fujii H., Uchida-Kobayashi S., Morikawa H., Tamori A. (2019). Association between HLA-DQA1/DRB1 Polymorphism and Development of Hepatocellular Carcinoma during Entecavir Treatment. J. Gastroenterol. Hepatol..

[8] Kozuka R., Enomoto M., Dong M.P., Hai H., Thuy L.T.T., Odagiri N., Yoshida K., Kotani K., Motoyama H., Kawamura E. (2022). Soluble Programmed Cell Death-1 Predicts Hepatocellular Carcinoma Development during Nucleoside Analogue Treatment. Sci. Rep..

[9] Kozuka R., Enomoto M., Yukawa-Muto Y., Odagiri N., Kotani K., Motoyama H., Kawamura E., Hagihara A., Fujii H., Uchida-Kobayashi S. (2024). Hepatitis B Surface Antigen Glycan Isomer Is a Predictor of the Development of Hepatocellular Carcinoma during Nucleoside/Nucleotide Analog Therapy. Hepatol. Res..

[10] Hosaka T., Suzuki F., Kobayashi M., Fujiyama S., Kawamura Y., Sezaki H., Akuta N., Kobayashi M., Suzuki Y., Saitoh S. (2022). Ultrasensitive Assay for Hepatitis B Core-Related Antigen Predicts Hepatocellular Carcinoma Incidences during Entecavir. Hepatol. Commun..

[11] Huang S.-C., Su T.-H. (2025). Divergent Roles of MASLD Components in Chronic Hepatitis B: A Double-Edged Sword. J. Hepatol..

[12] Fan R., Niu J., Ma H., Xie Q., Cheng J., Rao H., Dou X., Xie J., Zhao W., Peng J. (2021). Association of Central Obesity with Hepatocellular Carcinoma in Patients with Chronic Hepatitis B Receiving Antiviral Therapy. Aliment. Pharmacol. Ther..

[13] Haberl E.M., Weiss T.S., Peschel G., Weigand K., Köhler N., Pauling J.K., Wenzel J.J., Höring M., Krautbauer S., Liebisch G. (2021). Liver Lipids of Patients with Hepatitis B and C and Associated Hepatocellular Carcinoma. Int. J. Mol. Sci..

[14] Du Z., Yin S., Liu B., Zhang W., Sun J., Fang M., Xu Y., Hua K., Tu P., Zhang G. (2023). Metabolomics and Network Analysis Uncovered Profound Inflammation-Associated Alterations in Hepatitis B Virus-Related Cirrhosis Patients with Early Hepatocellular Carcinoma. Heliyon.

[15] Abel S., De Kock M., van Schalkwyk D.J., Swanevelder S., Kew M.C., Gelderblom W.C.A. (2009). Altered Lipid Profile, Oxidative Status and Hepatitis B Virus Interactions in Human Hepatocellular Carcinoma. Prostaglandins Leukot. Essent. Fatty Acids.

[16] Das U.N. (2019). Beneficial Role of Bioactive Lipids in the Pathobiology, Prevention, and Management of HBV, HCV and Alcoholic Hepatitis, NAFLD, and Liver Cirrhosis: A Review. J. Adv. Res..

[17] Hajeyah A.A., Griffiths W.J., Wang Y., Finch A.J., O’Donnell V.B. (2020). The Biosynthesis of Enzymatically Oxidized Lipids. Front. Endocrinol..

[18] Meng Y.-W., Liu J.-Y. (2024). Pathological and Pharmacological Functions of the Metabolites of Polyunsaturated Fatty Acids Mediated by Cyclooxygenases, Lipoxygenases, and Cytochrome P450s in Cancers. Pharmacol. Ther..

[19] Gong Z.-G., Zhao W., Zhang J., Wu X., Hu J., Yin G.-C., Xu Y.-J. (2017). Metabolomics and Eicosanoid Analysis Identified Serum Biomarkers for Distinguishing Hepatocellular Carcinoma from Hepatitis B Virus-Related Cirrhosis. Oncotarget.

[20] Lu Y., Fang J., Zou L., Cui L., Liang X., Lim S.G., Dan Y.Y., Ong C.N. (2018). Omega-6-Derived Oxylipin Changes in Serum of Patients with Hepatitis B Virus-Related Liver Diseases. Metabolomics.

[21] European Association for the Study of the Liver (2017). EASL 2017 Clinical Practice Guidelines on the Management of Hepatitis B Virus Infection. J. Hepatol..

[22] Terrault N.A., Lok A.S.F., McMahon B.J., Chang K.-M., Hwang J.P., Jonas M.M., Brown R.S., Bzowej N.H., Wong J.B. (2018). Update on Prevention, Diagnosis, and Treatment of Chronic Hepatitis B: AASLD 2018 Hepatitis B Guidance. Hepatology.

[23] Bedossa P., Poynard T. (1996). An Algorithm for the Grading of Activity in Chronic Hepatitis C. The METAVIR Cooperative Study Group. Hepatology.

[24] Hamaguchi M., Kojima T., Itoh Y., Harano Y., Fujii K., Nakajima T., Kato T., Takeda N., Okuda J., Ida K. (2007). The Severity of Ultrasonographic Findings in Nonalcoholic Fatty Liver Disease Reflects the Metabolic Syndrome and Visceral Fat Accumulation. Am. J. Gastroenterol..

[25] Snaebjornsson M.T., Janaki-Raman S., Schulze A. (2020). Greasing the Wheels of the Cancer Machine: The Role of Lipid Metabolism in Cancer. Cell Metab..

[26] Razdan A., Main N.M., Chiu V., Shackel N.A., de Souza P., Bryant K., Scott K.F. (2021). Targeting the Eicosanoid Pathway in Hepatocellular Carcinoma. Am. J. Cancer Res..

[27] Santiso A., Heinemann A., Kargl J. (2024). Prostaglandin E2 in the Tumor Microenvironment, a Convoluted Affair Mediated by EP Receptors 2 and 4. Pharmacol. Rev..

[28] Chen C., Guan J., Gu X., Chu Q., Zhu H. (2022). Prostaglandin E2 and Receptors: Insight into Tumorigenesis, Tumor Progression, and Treatment of Hepatocellular Carcinoma. Front. Cell Dev. Biol..

[29] Heinrich L., Booijink R., Khurana A., Weiskirchen R., Bansal R. (2022). Lipoxygenases in Chronic Liver Diseases: Current Insights and Future Perspectives. Trends Pharmacol. Sci..

[30] Xu X.-M., Yuan G.-J., Deng J.-J., Guo H.-T., Xiang M., Yang F., Ge W., Chen S.-Y. (2012). Inhibition of 12-Lipoxygenase Reduces Proliferation and Induces Apoptosis of Hepatocellular Carcinoma Cells in Vitro and in Vivo. Hepatobiliary Pancreat. Dis. Int..

[31] Ma J., Zhang L., Zhang J., Liu M., Wei L., Shen T., Ma C., Wang Y., Chen Y., Zhu D. (2013). 15-Lipoxygenase-1/15-Hydroxyeicosatetraenoic Acid Promotes Hepatocellular Cancer Cells Growth through Protein Kinase B and Heat Shock Protein 90 Complex Activation. Int. J. Biochem. Cell Biol..

[32] Okuno T., Yokomizo T. (2021). Metabolism and Biological Functions of 12(S)-Hydroxyheptadeca-5Z,8E,10E-Trienoic Acid. Prostaglandins Other Lipid Mediat..

[33] Yasukawa K., Okuno T., Ogawa N., Kobayashi Y., Yokomizo T. (2023). Identification and Characterization of Bioactive Metabolites of 12-Hydroxyheptadecatrienoic Acid, a Ligand for Leukotriene B4 Receptor 2. J. Biochem..

[34] Giusti F., Casiraghi M., Point E., Damian M., Rieger J., Bon C.L., Pozza A., Moncoq K., Banères J.-L., Catoire L.J. (2020). Structure of the Agonist 12-HHT in Its BLT2 Receptor-Bound State. Sci. Rep..

[35] Liu M., Saeki K., Matsunobu T., Okuno T., Koga T., Sugimoto Y., Yokoyama C., Nakamizo S., Kabashima K., Narumiya S. (2014). 12-Hydroxyheptadecatrienoic Acid Promotes Epidermal Wound Healing by Accelerating Keratinocyte Migration via the BLT2 Receptor. J. Exp. Med..

[36] Storniolo C.E., Pequera M., Company F., Moreno J.J. (2021). 12(S)-Hydroxyheptadeca-5Z,8E,10E-Trienoic Acid (12-HHT) Induces Cell Growth and Improves Barrier Function through BLT2 Interaction in Intestinal Epithelial Caco-2 Cell Cultures. Biochem. Pharmacol..

[37] Iizuka Y., Okuno T., Saeki K., Uozaki H., Okada S., Misaka T., Sato T., Toh H., Fukayama M., Takeda N. (2010). Protective Role of the Leukotriene B4 Receptor BLT2 in Murine Inflammatory Colitis. FASEB J..

[38] Yokomizo T. (2015). Two Distinct Leukotriene B4 Receptors, BLT1 and BLT2. J. Biochem..

[39] Daniel N., Genua F., Jenab M., Mayén A.-L., Chatziioannou A.C., Keski-Rahkonen P., Hughes D.J. (2024). The Role of the Gut Microbiome in the Development of Hepatobiliary Cancers. Hepatology.

[40] Schneider K.M., Mohs A., Gui W., Galvez E.J.C., Candels L.S., Hoenicke L., Muthukumarasamy U., Holland C.H., Elfers C., Kilic K. (2022). Imbalanced Gut Microbiota Fuels Hepatocellular Carcinoma Development by Shaping the Hepatic Inflammatory Microenvironment. Nat. Commun..

[41] Dapito D.H., Mencin A., Gwak G.-Y., Pradere J.-P., Jang M.-K., Mederacke I., Caviglia J.M., Khiabanian H., Adeyemi A., Bataller R. (2012). Promotion of Hepatocellular Carcinoma by the Intestinal Microbiota and TLR4. Cancer Cell.

[42] Sterling R.K., Patel K., Duarte-Rojo A., Asrani S.K., Alsawas M., Dranoff J.A., Fiel M.I., Murad M.H., Leung D.H., Levine D. (2025). AASLD Practice Guideline on blood-based noninvasive liver disease assessment of hepatic fibrosis and steatosis. Hepatology.

[43] Suh B., Park S., Shin D.W., Yun J.M., Yang H.-K., Yu S.J., Shin C.-I., Kim J.-S., Ahn E., Lee H. (2015). High Liver Fibrosis Index FIB-4 Is Highly Predictive of Hepatocellular Carcinoma in Chronic Hepatitis B Carriers: SUH, PARK ET AL. Hepatology.

[44] Inoue J., Akahane T., Kobayashi T., Kimura O., Sato K., Ninomiya M., Iwata T., Takai S., Kisara N., Sato T. (2024). Usefulness of the Fibrosis-4 Index and Alanine Aminotransferase at 1 Year of Nucleos(t)Ide Analog Treatment for Prediction of Hepatocellular Carcinoma in Chronic Hepatitis B Patients. Hepatol. Res..

